# HIV infection drives interferon signaling within intestinal SARS-CoV-2 target cells

**DOI:** 10.1172/jci.insight.148920

**Published:** 2021-08-23

**Authors:** Rabiah Fardoos, Osaretin E. Asowata, Nicholas Herbert, Sarah K. Nyquist, Yenzekile Zungu, Alveera Singh, Abigail Ngoepe, Ian M. Mbano, Ntombifuthi Mthabela, Dirhona Ramjit, Farina Karim, Warren Kuhn, Fusi G. Madela, Vukani T. Manzini, Frank Anderson, Bonnie Berger, Tune H. Pers, Alex K. Shalek, Alasdair Leslie, Henrik N. Kløverpris

**Affiliations:** 1 Africa Health Research Institute, Durban, South Africa.; 2 Department of Immunology and Microbiology, University of Copenhagen, Copenhagen, Denmark.; 3 School of Laboratory Medicine and Medical Sciences, University of KwaZulu-Natal, Durban, South Africa.; 4 Institute for Medical Engineering & Science, Department of Chemistry, and Koch Institute for Integrative Cancer Research, Massachusetts Institute of Technology (MIT), Cambridge, Massachusetts, USA.; 5 Broad Institute of MIT and Harvard, Cambridge, Massachusetts, USA.; 6 Ragon Institute of MGH, MIT and Harvard, Cambridge, Massachusetts, USA.; 7 Program in Computational and Systems Biology, MIT, Cambridge, Massachusetts, USA.; 8 ENT Department, General Justice Gizenga Mpanza Regional Hospital (Stanger Hospital), University of KwaZulu-Natal, Durban, South Africa.; 9 Discipline of General Surgery, Inkosi Albert Luthuli Central Hospital, University of KwaZulu-Natal, Durban, South Africa.; 10 Computer Science and Artificial Intelligence Laboratory and Department of Mathematics, MIT, Cambridge, Massachusetts, USA.; 11 Novo Nordisk Foundation Center for Basic Metabolic Research, Faculty of Health and Medical Sciences, University of Copenhagen, Copenhagen, Denmark.; 12 Division of Infection and Immunity, University College London, London, United Kingdom.

**Keywords:** AIDS/HIV, COVID-19, Innate immunity

## Abstract

SARS-CoV-2 infects epithelial cells of the human gastrointestinal (GI) tract and causes related symptoms. HIV infection impairs gut homeostasis and is associated with an increased risk of COVID-19 fatality. To investigate the potential link between these observations, we analyzed single-cell transcriptional profiles and SARS-CoV-2 entry receptor expression across lymphoid and mucosal human tissue from chronically HIV-infected individuals and uninfected controls. Absorptive gut enterocytes displayed the highest coexpression of SARS-CoV-2 receptors *ACE2*, *TMPRSS2*, and *TMPRSS4*, of which *ACE2* expression was associated with canonical interferon response and antiviral genes. Chronic treated HIV infection was associated with a clear antiviral response in gut enterocytes and, unexpectedly, with a substantial reduction of *ACE2* and *TMPRSS2* target cells. Gut tissue from SARS-CoV-2–infected individuals, however, showed abundant SARS-CoV-2 nucleocapsid protein in both the large and small intestine, including an HIV-coinfected individual. Thus, upregulation of antiviral response genes and downregulation of *ACE2* and *TMPRSS2* in the GI tract of HIV-infected individuals does not prevent SARS-CoV-2 infection in this compartment. The impact of these HIV-associated intestinal mucosal changes on SARS-CoV-2 infection dynamics, disease severity, and vaccine responses remains unclear and requires further investigation.

## Introduction

Gastrointestinal (GI) tract symptoms are observed in up to 60% of COVID-19 patients and precede respiratory symptoms (1). SARS-CoV-2 can be detected within intestinal tissues (2, 3), and about 30% of COVID-19 patients harbor detectable viral RNA in their stool (4), which is associated with more severe GI symptoms (1). The observed GI disorders include vomiting, nausea, and diarrhea; can manifest local and systemic disease; and may lead to fecal-oral transmission of virus (2, 5, 6). High expression of ACE2, the primary receptor for SARS-CoV-2, is found on the luminal surface of differentiated small intestinal epithelial cells, whereas crypt-based cells express lower levels (7, 8). Studies of human small intestinal organoids show that the mature enterocytes are the major source for SARS-CoV-2 replication (2, 6, 9). These cells coexpress the serine proteases TMPRSS2 and TMPRSS4, which promote SARS-CoV-2 spike protein fusion and viral entry into enterocytes (6).

HIV infection in the GI tract results in rapid and massive CD4^+^ T cell depletion, with associated changes in the microbiome and elevated translocation of microbial products across the epithelial barrier. These events precipitate a so-called leaky gut syndrome, which is thought to be central to HIV pathology (10–13). The elevated systemic immune activation, inflammatory responses, and gut dysbiosis associated with this syndrome (14) may influence the overall type I interferon responses, and therefore compromise both local and systemic responses to SARS-CoV-2 infection, including responses within the lung mucosa (15). Multiple studies prior to the SARS-CoV-2 pandemic have demonstrated that HIV-induced systemic immune activation renders HIV-infected individuals vulnerable to airborne infections, such as tuberculosis (16, 17). Recent data suggest that SARS-CoV-2 and HIV coinfected individuals overall have a 2.1-fold increased risk of dying from COVID-19 (16), with a risk factor reaching more than 3.5 in viremic individuals with CD4 count below 200 cells/mm^3^ (18). This is consistent with low nadir CD4^+^ T cell counts being associated with increased mortality in COVID-19 patients (19). Therefore, understanding the dynamics of SARS-CoV-2 infection in the gut of HIV-infected individuals is a pressing area of research.

Single-cell transcriptomic analysis from different tissues reported increased coexpression of *ACE2* and *TMPRSS2* transcripts in the ileum of SHIV-infected nonhuman primates and within the lung of HIV-infected humans compared with uninfected controls (20), suggesting a potential higher risk of SARS-CoV-2 infection in HIV-infected individuals. One of the isoforms of *ACE2*, encoded by exon 1c (*dACE2*), has recently been confirmed as an interferon-stimulating gene (ISG) and responds directly to type I interferons and SARS-CoV-2 infection in the small intestine (21). Therefore, type 1 interferon stimulation and altered regulation of *ACE2* could be exploited by SARS-CoV-2 to enhance infection (20). However, no human studies have determined the impact of HIV infection on SARS-CoV-2 entry receptors in the gut, or on the transcriptional landscape of the gut enterocytes expressing them.

To explore the potential impact of HIV infection on gut epithelial cells susceptible to SARS-CoV-2 infection, we investigated the gene expression profile of *ACE2*, *TMPRSS2*, and *TMPRSS4* using single-cell RNA-Seq (scRNA-Seq) data sets from human SARS-CoV-2–uninfected tonsil, liver, lymph node, duodenum, and blood, as well as a published human lung scRNA-Seq data set (20). All study participants were from clinics in KwaZulu-Natal, South Africa, recruited within extremely high HIV-1–endemic areas. Our data show that *ACE2* was associated with interferon response genes in HIV-uninfected individuals, supporting the hypothesis that *ACE2* expression is linked with interferon signaling within gut enterocytes. In addition, and as expected, we show that HIV infection itself drove a strong interferon signaling response in these cells. Surprisingly, however, HIV infection was associated with a downregulation of *ACE2* expression in all cell types studied. These data suggest that, although ACE2 is associated with interferon signaling genes, the isoforms detected here are differentially regulated from canonical interferon signaling genes. The reduction in potential target cells may affect infectability, propagation of infection of SARS-CoV-2 in HIV-infected individuals, and local and systemic immunity beyond the gut, such as the lung mucosa (15). Nevertheless, using gut biopsies from coinfected individuals, we observed the presence of SARS-CoV-2 and HIV proteins in gut enterocytes, indicating this compartment remains vulnerable to infection.

## Results

### SARS-CoV-2 host entry receptors are enriched in the human small intestine.

**Figure 1:**
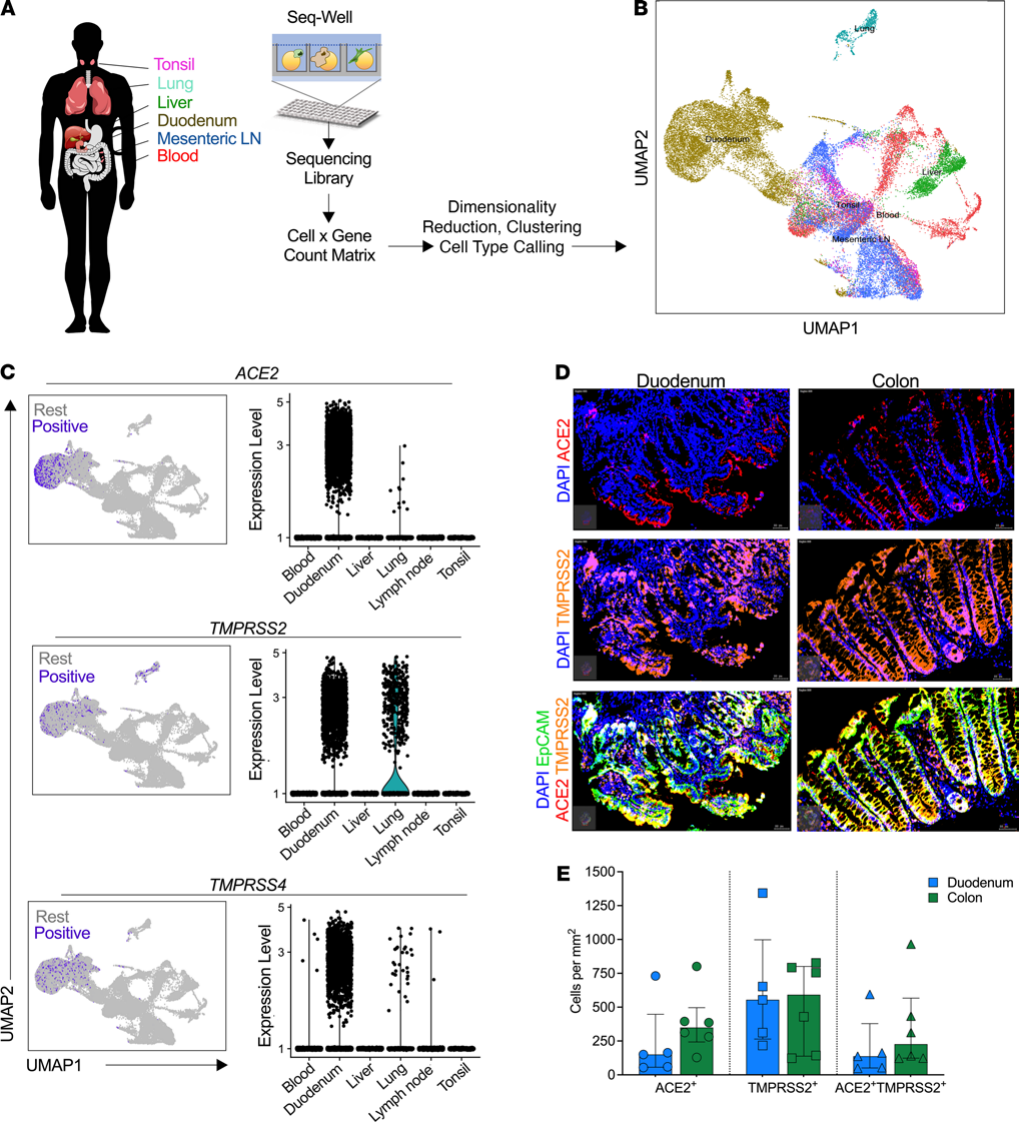
SARS-CoV-2 putative target cells are enriched in the human duodenum. (**A**) Schematic of protocol for isolation of different tissues for scRNA-Seq using Seq-Well S^3^, to identify cell types. (**B**) Uniform manifold approximation and projection (UMAP) of 32,381 cells colored by tissue source. (**C**) Left: UMAP of epithelial cells showing expression of *ACE2* (top), *TMPRSS2* (middle), and *TMPRSS4* (bottom) among all tissue sources from human donors. Color coding is as follows: purple, RNA positive; gray, RNA negative. Right: Corresponding violin plots of expression values for *ACE2* (top), *TMPRSS2* (middle), and *TMPRSS4* (bottom). (**D**) Representative fluorescence immunohistochemistry image of gut tissue showing *ACE2* (red), *TMPRSS2* (orange), EpCAM (green), and DAPI (blue) of duodenum and colon. Bars: 20 μm for all images. (**E**) Quantification of *ACE2* and *TMPRSS2* proportion of total cells stained with EpCAM. Data shown as median ± SD.

SARS-CoV-2 infection of human cells requires surface expression of the primary receptor *ACE2* and one of the coreceptors *TMPRSS2* (22) or *TMPRSS4* (3, 6). scRNA-Seq analysis showed that *ACE2* expression is primarily restricted to type II pneumocytes in the lung, gut absorptive enterocytes, and goblet secretory cells of the nasal mucosa (20). In this study, we applied high-throughput scRNA-Seq (23, 24) to profile distinct human tissue samples (Figure 1A) and analyzed 32,381 cells from blood, tonsil, lung, duodenum, and mesenteric lymph node (lung data set has been previously described in ref. 20) (Figure 1B), for expression of genes encoding *ACE2*, *TMPRSS2*, and *TMPRSS4*. We identified 18 distinct cellular subclusters in this data set (Supplemental Figure 1A; supplemental material available online with this article; https://doi.org/10.1172/jci.insight.148920DS1), with the majority of cells expressing *ACE2*, *TMPRSS2*, and *TMPRSS4* located within duodenum and to a lesser extent in lung tissue and with no expression in the other tissues analyzed (Figure 1C). We next used fluorescence immunohistochemistry to costain epithelial cells (EpCAM) for *ACE2* and *TMPRSS2* and found distinct in situ expression patterns between the small and large intestine (Figure 1D and Supplemental Figure 1B). The duodenum tissue expressed high levels of *ACE2* facing outward toward the lumen side, whereas the colon tissue was characterized by *ACE2* expression at the bottom half of the crypt. *TMPRSS2* was expressed in most of the epithelial cells and was coexpressed with *ACE2* in both duodenum and colon tissue. We found no quantitative differences in protein expression levels between both compartments for *ACE2* and *TMPRSS2* (Supplemental Table 1 and Figure 1E). High SARS-CoV-2 entry receptor expression in the small intestine is consistent with recent reports (2, 5, 6, 20) and with detection of SARS-CoV-2 nucleocapsid in the small intestine of infected individuals (3). Locational differences in *ACE2* expression between the duodenum and colon may be linked to the physiology of *ACE2* in these compartments, such as regulation of intestinal inflammation and amino acid homeostasis (7, 8), and may influence SARS-CoV-2 infection dynamics in each compartment.

### ACE2 expression is dominated by absorptive enterocytes.

**Figure 2:**
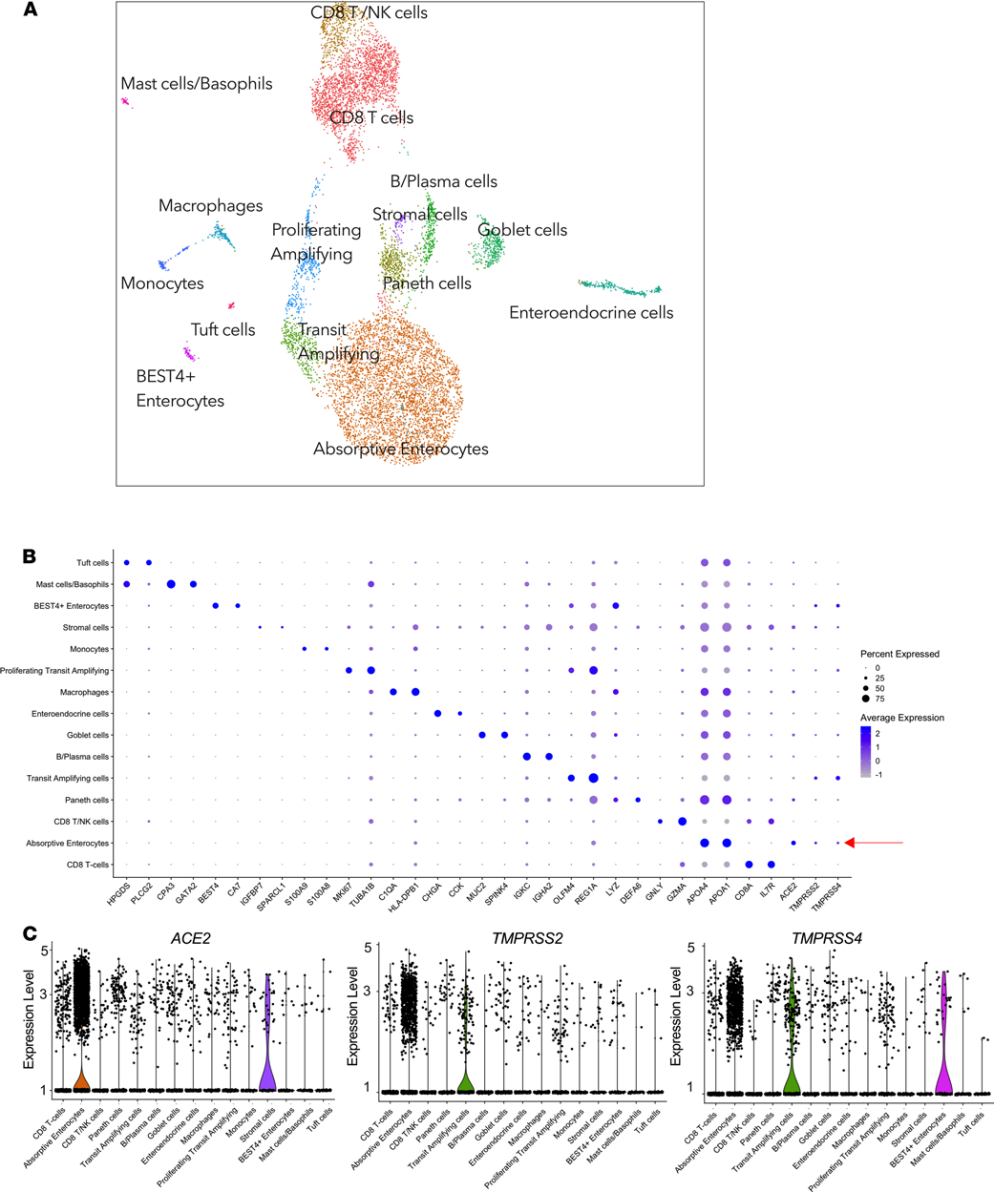
*ACE2* expression is enriched in absorptive enterocytes. (**A**) UMAP of 13,056 cells from endoscopic pinch biopsies, colored by cell type. (**B**) Dot plot of 2 defining genes for each cell type and *ACE2*, *TMPRSS2*, and *TMPRSS4*. Dot size represents fraction of cells within cell type expressing a given gene, and color intensity represents binned count-based expression amounts (log_scaled UMI+1_) among expressing cells. Red arrow indicates cell type with largest proportion of *ACE2^+^TMPRSS2^+^TMPRSS4^+^* cells; full results can be found in Supplemental Table 2A. (**C**) Expression of *ACE2* (left), *TMPRSS2* (middle), and *TMPRSS4* (right) among all subsets from duodenum. UMI, unique molecular identifier.

Next, focusing on the small intestine only, for which most samples were available, we evaluated the expression of *ACE2*, *TMPRSS2*, *TMPRSS4*, and other transmembrane serine proteases in duodenal cellular subsets using scRNA-Seq data from 4 HIV-uninfected female donors (Table 1 and Supplemental Table 1). To assign cellular identity, we performed variable gene selection, performed dimensionality reduction by UMAP with graph-based clustering (Figure 2A), created a cells-by-genes expression matrix (Figure 2B), and identified 15 distinct major cell clusters from 13,056 duodenum cells. Cell cluster identities were assigned based on the most highly differentially expressed genes (DEGs) for each cluster (Figure 2B and Supplemental Table 2A). This approach identified absorptive enterocytes as the predominant *ACE2*-expressing cell type, defined by expression of *APOA1* and *APOA4* (41.3% *ACE2*^+^, FDR-adjusted *P* = 1.01 × 10^–227^) (Figure 2B). These cells also more frequently expressed *TMPRSS2* and *TMPRSS4* than any of the other subsets (Figure 2C and Supplemental Figure 2A). The majority of absorptive enterocytes also expressed *ST14* and *TMPRSS15* and to a much lesser extent *TMPRSS3* and *TMPRSS6* (Supplemental Figure 2B). There is no known role for these other serine proteases in SARS-CoV-2 infection, but it does imply the importance of this family of molecules to the biology of absorptive enterocytes (25). Focusing on epithelial cells, we consistently identified absorptive enterocytes as the main source of *ACE2*, *TMPRSS2*, and *TMPRSS4* gene expression (Supplemental Figure 3, A–D) and SARS-CoV-2 putative target cells due to coexpression of these markers: *ACE2^+^TMPRSS2^+^* (412 cells, 12% of all epithelial cells), *ACE2*^+^*TMPRSS4^+^* subset (392 cells, 11% of all epithelial cells), and a smaller subset coexpressing all 3 genes *ACE2^+^TMPRSS2^+^TMPRSS4^+^* (154 cells, 5%; Supplemental Figure 3E). Using a Monocle single-cell trajectory analysis, we uncovered a clear potential differentiation trajectory from “intestinal stem cell–like” cells (*LGR5*, *CD44*, *EPHB2*), through transit amplifying cells (*OLFM4*) and Paneth cells (*DEFA5*, *DEFA6*), to the dominant absorptive enterocyte clusters (*APOA1*, *APOA4*) enriched for putative SARS-CoV-2–susceptible cells (Supplemental Figure 4), suggesting that terminal differentiated cells are likely to be the most susceptible to infection. Thus, cellular differentiation was linked to SARS-CoV-2 entry receptor expression. Overall, these observations are in line with previous reports indicating that *TMPRSS2* and *TMPRSS4* are important coreceptors for SARS-CoV-2 fusion and entry into *ACE2*^+^ differentiated enterocytes in the human duodenum and small intestine (6, 26).

### ACE2 expression is associated with ISGs.

**Figure 3:**
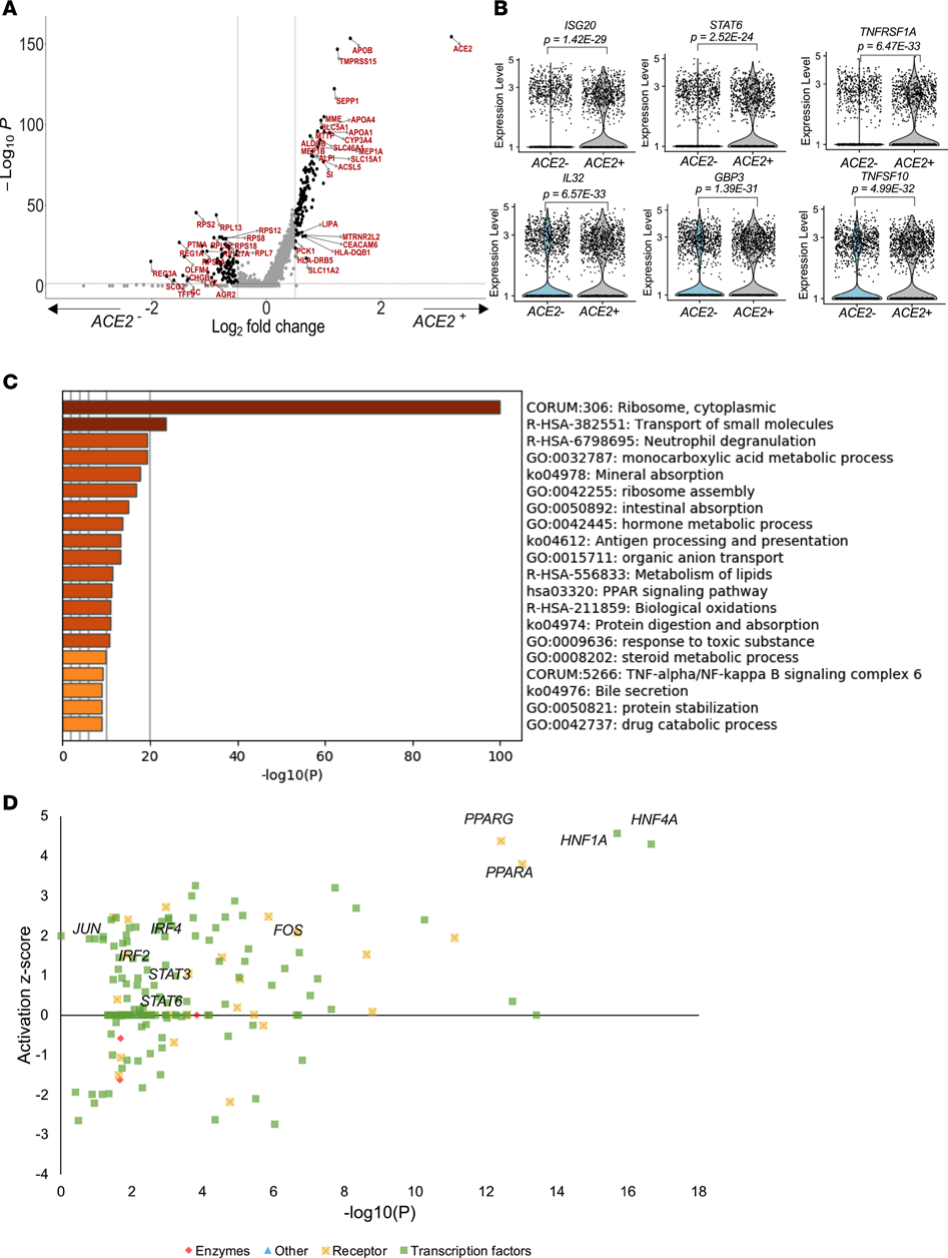
*ACE2*-expressing absorptive enterocytes are linked to ISGs and functional absorptive pathways. (**A**) Volcano plot of DEGs (Supplemental Table 2B) within epithelial cells from HIV-uninfected individuals (*n* = 4) highlighting genes with more than 0.5 fold change and adjusted *P* < 5.0 × 10^8^. (**B**) Violin plots of genes differentially expressed among *ACE2^+^* and *ACE2*^−^ epithelial cells, FDR-adjusted *P* < 0.05; full results can be found in Supplemental Table 2B. (**C**) GO BP enrichment analysis of the DEGs from epithelial cell analysis upregulated in *ACE2^+^* compared with *ACE2^–^*. *P* value was derived by a hypergeometric test. (**D**) Selected upstream drivers of pathways shown in **C** from DEGs in Supplemental Table 2D. GO, Gene Ontology; BP, biological pathway.

Next, we compared *ACE2*-expressing (*ACE2^+^*) with nonexpressing (*ACE2^–^*) cells and identified 381 DEGs (*P* < 0.05) (Supplemental Table 2B). The majority of these genes were upregulated together with APOB and APOA4, confirming that *ACE2* expression is associated with absorptive enterocytes (Figure 3A). In addition, these DEGs included a range of known interferon response genes, such as *ISG20*, which interferes with viral replication (27); *STAT6*, which is important for immune signals emanating from interleukin-4 receptors (28); *TNFSF10*, a proapoptotic cytokine (29, 30); *TNFRSF1A* involved in TNF-α signaling; and the proinflammatory cytokine *IL-32*, which is involved in the pathogenesis and progression of a number of inflammatory disorders (31) (Figure 3B). In support of the cell subsetting approach, pathway analysis of *ACE2^+^* cells was consistent with functional absorptive cells, including pathways involved in transport of small molecules, mineral absorption, intestinal absorption, bile secretion, etc. (Figure 3C). Further subsetting on *ACE2^+^TMPRSS2^+^* cells as putative SARS-CoV-2 targets and comparison with the remaining epithelial cells (Supplemental Table 2C) showed significant upregulation of *IFNGR1* within *ACE2^+^TMPRSS2^+^* cells (Supplemental Table 2C), further supporting the expression of ISG signatures in susceptible target cells. Analysis of potential upstream drivers of the *ACE2*-associated DEGs identified canonical ISG genes, such as *STAT3*, *IRF2*, and *IRF4* (Figure 3D and Supplemental Table 2D). These data show that epithelial cells, and particularly the absorptive enterocytes, are enriched for *ACE2*, *TMPRSS2*, and *TMPRSS4* expression and that *ACE2* expression in these cells is upregulated in conjunction with known ISGs.

### HIV infection drives interferon response in gut absorptive enterocytes.

**Figure 4:**
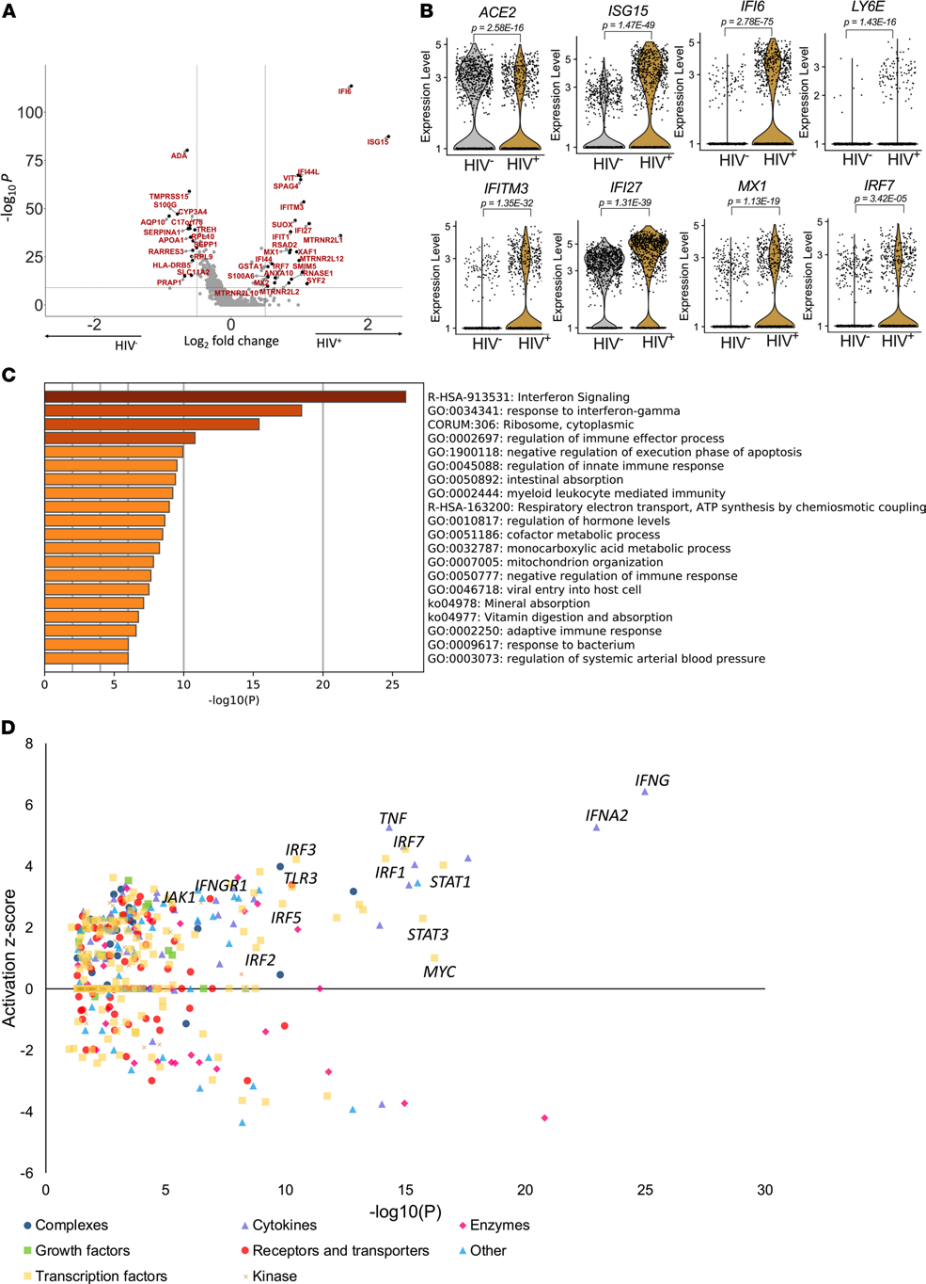
HIV infection downregulates *ACE2* expression and drives interferon signaling in absorptive enterocytes. (**A**) Volcano plot of DEGs (Supplemental Table 3B) within absorptive enterocytes in HIV-infected and HIV-uninfected cells highlighting genes with more than 0.5-fold change and adjusted *P* < 5.0 × 10^8^. (**B**) Violin plots of expression of *ACE2* and interferon-responsive genes among absorptive enterocytes from HIV^–^ (*n* = 4) and HIV^+^ART^+^ (*n* = 5). (**C**) GO BP enrichment analysis of the DEGs of absorptive enterocytes upregulated in HIV^–^ (*n* = 4) and HIV*^+^*ART*^+^* (*n* = 5). *P* value was derived by a hypergeometric test. (**D**) Activation *z* score of upstream drivers from DEGs shown in **A** and Supplemental Table 3D color-coded by their functional categories.

HIV infection depletes CD4^+^ T cells in the gut and has a major impact on the barrier integrity (10, 12), and the intestinal epithelium is dominated by absorptive enterocytes with high expression levels of SARS-CoV-2 entry receptors compared with other epithelial subsets (see Supplemental Figure 3 and Supplemental Table 3A). Therefore, we next determined the influence of HIV infection on potential SARS-CoV-2 target cells, by comparing the gene expression of cells from duodenal tissues collected from individuals with chronic HIV infection on long-term antiretroviral therapy (ART) and HIV-uninfected participants. We found 160 DEGs (134 upregulated and 26 downregulated; Figure 4A and Supplemental Table 3B). Consistent with the gut mucosal inflammation associated with HIV (32), these included strong upregulation of numerous canonical ISG and antiviral genes, including *ISG15*, *IFI6*, *LY6E*, *IFITM3*, *IFI27*, *MX1*, and *IRF7* (Figure 4B and Supplemental Table 3B). In contrast to earlier studies (20), and despite the observed interferon signaling, *ACE2* expression was significantly reduced in enterocytes isolated from subjects with treated chronic HIV infection (Figure 4B), suggesting that *ACE2* expression itself may not directly act as an ISG (21). Pathway analysis of DEGs in absorptive enterocytes showed that HIV infection was associated with a profound upregulation of interferon signaling (Figure 4C). Analysis of potential upstream drivers of these DEGs indicated type I interferon and multiple interferon response factors, consistent with a constitutive antiviral response program within absorptive enterocytes that was induced by HIV infection despite ART (Figure 4D and Supplemental Table 3C). Finally, subsetting on the remaining *ACE2*-expressing epithelial cells only showed a consistent strong upregulation of these canonical ISGs in HIV-infected subjects (Supplemental Figure 5, A and B, and Supplemental Table 3D). Taken together, these data show that, despite suppression of plasma viremia, HIV infection induces a strong interferon antiviral response in gut enterocytes in general and also within *ACE2*-expressing putative SARS-CoV-2 target cells. However, this does not drive *ACE2* expression itself, which is reduced in HIV-infected individuals.

### Reduced numbers of ACE2-expressing absorptive enterocytes and SARS-CoV-2 target cells in HIV-infected individuals.

**Figure 5:**
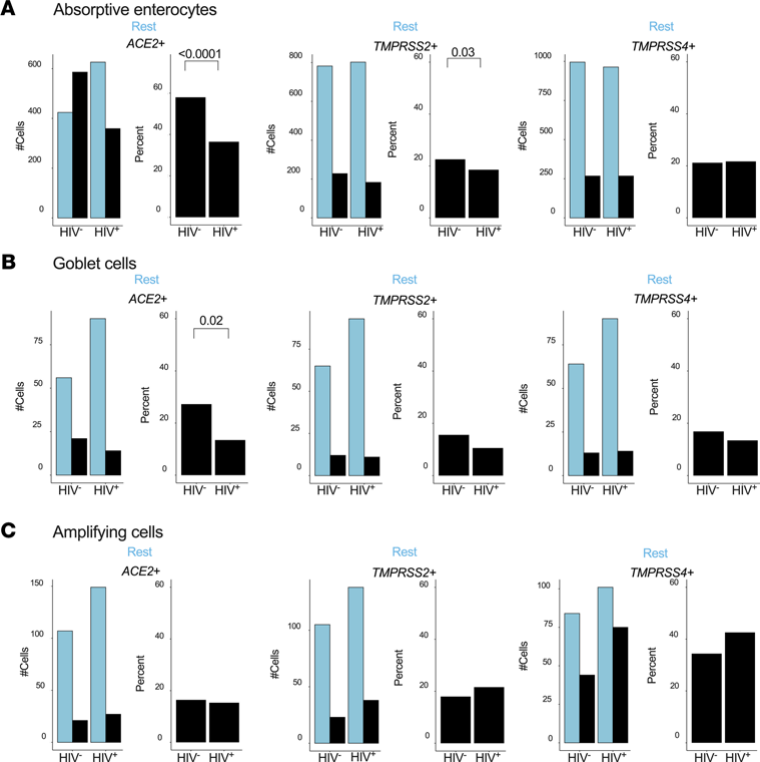
HIV infection reduces the frequency of SARS-CoV-2 putative target cells within the small intestine. (**A**) Actual number of absorptive enterocytes (left) and percentage expression (right) of *ACE2*, *TMPRSS2*, and *TMPRSS4* by HIV status. (**B**) Number of goblet cells (left) and percentage (right) expressing *ACE2*, *TMPRSS2*, and *TMPRSS4* by HIV status. (**C**) Number of transit-amplifying cells (left) and percentage (right) expressing *ACE2*, *TMPRSS2*, and *TMPRSS4* by HIV status. *P* values by Fisher’s Exact Test. Rest, cells not expressing the indicated transcript.

Having observed an unexpected downregulation of *ACE2* in HIV-infected subjects, we quantified the number of epithelial cells susceptible to SARS-CoV-2 infection and compared the relative frequencies of absorptive enterocytes, goblet cells, and transit-amplifying cells (see Figure 2) between HIV-infected and uninfected individuals. Overall, the numbers of *ACE2*- and *TMPRSS2*-expressing absorptive enterocytes were significantly reduced in HIV infection, while *TMPRSS4* was unaffected (Figure 5A). The same trend was observed for goblet cells but not for transit-amplifying cells (Figure 5, B and C). When we analyzed absorptive enterocytes expressing 2 or more entry receptors, we found a similar significant reduction of all combinations of SARS-CoV-2 putative target cells, with the same trend for goblet cells but not for transit-amplifying cells (Supplemental Figure 6). Thus, chronic HIV infection appears to reduce the total frequency of SARS-CoV-2 putative target cells within the small intestine.

### Abundant SARS-CoV-2 detection in small and large intestine irrespective of HIV coinfection.

**Figure 6:**
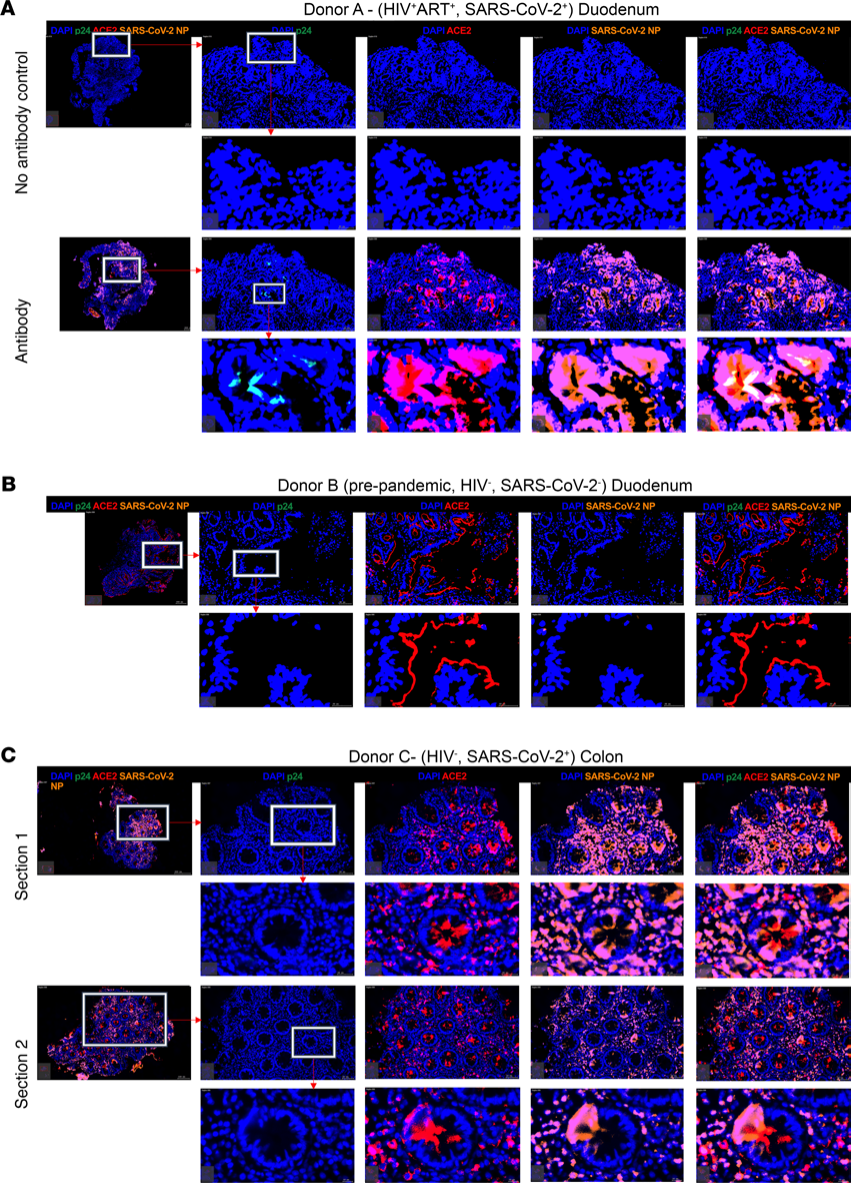
SARS-CoV-2 nucleocapsid detection overlaps with ACE2 expression in the small and large intestine. Representative fluorescence immunohistochemistry (F-IHC) images of duodenum and colon tissues showing HIV-p24 (green), *ACE2* (red), SARS-CoV-2 nucleocapsid protein (orange), and DAPI (blue). (**A**) F-IHC image of a duodenum tissue from an HIV^+^SARS-CoV-2^+^ participants by PCR including no antibody control (top). (**B**) F-IHC image of a duodenum tissue from an HIV^-^ SARS-CoV-2^+^ participant. (**C**) F-IHC image of a colon tissue from HIV^-^SARS-CoV-2^+^ with 2 sections shown from the same biopsy tissue. Scale bars are shown at the bottom right of each image. Scale bars: 20 μm and 100 μm for magnified inserts and main images, respectively.

Finally, to determine if HIV-associated loss of SARS-CoV-2 target cells and upregulation of antiviral genes prevented infection of the GI tract, we collected gut tissue from confirmed SARS-CoV-2 antigen PCR-positive participants with and without HIV infection. From a duodenum biopsy obtained from a SARS-CoV-2 and HIV coinfected individual on ART and with undetectable plasma HIV viremia (Supplemental Table 1), we identified abundant expression of SARS-CoV-2 nucleocapsid protein (NP) within the epithelial layer and colocalized with that of ACE2 entry receptor (Figure 6A). We found small, but detectable, levels of HIV-p24 protein in the same area but in cells that did not express ACE2, consistent with distinct viral entry receptor usage between SARS-CoV-2 and HIV infection. We repeated this staining in a pre-pandemic control sample and observed no SARS-CoV-2 NP staining (Figure 6B). Histology of colon samples collected from an HIV-uninfected SARS-CoV-2 PCR-positive donor also revealed abundant SARS-CoV-2 NP expression within the epithelial tissue overlapping with ACE2 (Figure 6C). These data confirm the presence of high levels of SARS-CoV-2 virus production in the GI tract (3) and show it is likely to occur in HIV-infected individuals despite upregulation of antiviral immunity and a loss of putative target cells in the small intestine.

## Discussion

In this study we performed single-cell transcriptomic profiling across different human tissue sites and identified high expression of SARS-CoV-2 entry receptors within absorptive enterocytes from the small intestine that we confirmed by in situ protein staining. We detected overlapping expression of SARS-CoV-2 NP and ACE2 within both the small and large intestine of SARS-CoV-2–infected individuals, confirming the infectability of these cells in vivo. *ACE2*, *TMPRSS2*, and *TMPRSS4* expression was highest in the duodenum followed by the lung, with little or no expression detected in the tonsil, liver, lymph node, and blood, consistent with published studies (20, 25, 33, 34). We found that ACE2 protein expression was restricted to the luminal region of the enterocytes in the duodenum, whereas in the colon, ACE2 was located closer to the crypt base (7, 8). This distinct location may be explained by differences in the physiological processes within these compartments where luminal duodenal ACE2 is reported to be important in amino acid transport and protein synthesis (35, 36), consistent with our pathway analysis of *ACE2*-expressing duodenal epithelial subsets (see Figure 3C).

In HIV-uninfected subjects, *ACE2* expression in the small intestine was associated with genes involved in interferon signaling, in agreement with recent observations and experimental data demonstrating upregulation of ACE2 in response to interferon signaling (20). However, more recently investigators have established that it is the truncated isoform of ACE2, *dACE2*, that is most likely to act as an ISG and not ACE2. This study found that *dACE2* was directly upregulated by both interferon stimulation and SARS-CoV-2 infection within human intestinal organoid cells, but the full-length *ACE2* was not (21). Importantly, *dACE2* does not function as a SARS-CoV-2 entry receptor (37). In the future, it would be interesting to also sequence the gut virome of these individuals to determine how this may contribute to interferon signaling within the intestinal mucosa. The transcriptomic profile of enterocytes from chronic HIV-infected individuals was characterized by a strong interferon signaling pathway that included upregulation of canonical ISGs such as *ISG15* and *IFI2*7 predicted to be driven by type I and II interferons. These data highlight the impact from HIV infection on the small intestine and contribute to understanding of mechanisms underlying the functional consequences in gut barrier integrity and overall pathology (10–13). The reduced frequency of *ACE2*-expressing cells in the intestinal mucosa was therefore unexpected. Both *ACE2* and canonical ISGs’ (*STAT1* and *IFI6*) levels remained elevated in absorptive enterocytes from nonhuman primates (NHPs) with treated SIV infection (20). Indeed, *IFI6* was also highly upregulated in HIV-infected subjects in our cohort (Figure 3B). Why *ACE2*-expressing cells were reduced is not clear from this study. Changes in the microbiome, however, have recently been shown to alter *ACE2* expression levels and disrupt the ACE/ACE2 axis, and HIV is known to cause gut dysbiosis (38, 39). It will therefore be interesting to investigate the link between HIV infection, altered microbiomes, and ACE2 expression levels in the gut of HIV-infected individuals. The Monocle lineage analysis conducted here suggests that ACE2 expression may be upregulated as enterocytes progress toward terminal differentiation. Therefore, a reduction of ACE2-expressing cells could result from interference in this process or in increased cell death of terminally differentiated ACE2-expressing cells. Individuals in this study were all women on long-term, fully suppressive ART, which may distinguish them somewhat from the experimentally infected NHPs, which were treated for 6 months. In addition, the impact of the gut microbiome on ACE2 expression in the small intestine, discussed above, may affect comparisons between experimental NHP studies and human cohorts (38, 39). We only used women for the transcriptional data in this study to avoid sex-biased gene expression, and therefore extending these gene expression profiles beyond women will require further validation. However, the persistent interferon signature in these women clearly implies that upstream drivers, such as type I and II interferons, have not diminished.

Although the implications for infectability of the gut mucosa for the SARS-CoV-2 virus remain unclear, our data from HIV and SARS-CoV-2 coinfected participants from whom we obtained ex vivo duodenum biopsies showed that SARS-CoV-2 infection certainly can occur in the gut of HIV-infected individuals. Whether HIV-infected individuals have longer SARS-CoV-2 sequelae from the gut (3) would be interesting to study in a large coinfected gut biopsy cohort, as would the impact of variant viruses, which may have different affinities for cell entry receptors (40, 41).

Although the literature is still emerging, in general, studies have observed that COVID-19 patients with controlled HIV coinfection and preserved CD4^+^ T cell counts have similar clinical trajectories to those without HIV infection (18, 42, 43). By contrast, immune-compromised, HIV-infected individuals with CD4^+^ T cell counts below 200 cells/mm^3^ are associated with increased COVID-19 disease severity and mortality (16, 18). Whether reduction of potential SARS-CoV-2 target cells in gut mucosa of HIV-infected subjects limits the additional effects of SARS-CoV-2 infection in this compartment warrants further studies. Further intestinal sampling from coinfected individuals is currently being sought to address this urgent question. Indeed, although the actual number of SARS-CoV-2 target cells was reduced, interferon signaling and pathways of metabolic and absorptive changes observed here in HIV-infected subjects prior to SARS-CoV-2 infection may still exacerbate disease or limit immunity in some individuals. Whether increased transmissibility of new SARS-CoV-2 variants, including beta and the recent dominant delta variants identified in high HIV prevalence populations in South Africa (44, 45), alter the GI tract–related symptoms and overall COVID-19 disease severity is also currently unknown. Finally, potentially altered induction of type I interferons by evolving SARS-CoV-2 variants could potentially also be linked to signaling within intestinal enterocytes and should be explored further.

## Methods

### Study participants.

Patients presenting to the GI surgical unit of Inkosi Albert Luthuli Central Hospital were recruited into this study after they provided written informed consent. Tonsil, liver, gut lymph node, duodenum, and colon biopsies with participant-matched blood samples were obtained during surgical procedures. Clinical information, including HIV status and demographic details of these participants, was collected using a structured questionnaire. HIV status was confirmed using the Determine HIV 1/2 Set (Abbott Laboratories) and COBAS TaqMan HIV-1 Test (Roche).

### Sample processing.

Mononuclear cells were isolated from blood, tonsil, liver, lymph node, and pooled duodenum pinches to average over individual pinch variation. Blood was collected in BD vacutainers with sodium heparin. Peripheral blood mononuclear cells were isolated using the Ficoll-Histopaque 1077 (MilliporeSigma) density gradient centrifugation. Duodenum and colon pinch biopsies (2 to 4 pinches) were removed by the operating GI surgeon and transported to the laboratory in cold PBS (pH 7.2). The PBS was decanted from the tubes containing the gut biopsies, which are about 5–8 mm in size, and they were incubated in epithelial strip buffer (PBS, 0.5 M EDTA, 1 M DTT, FBS, and penicillin/streptomycin) in a 37°C water bath for 10 minutes, with occasional agitation. Thereafter, the epithelial strip buffer was removed, and the tissues were digested in a buffer containing collagenase-D (0.5 mg/mL; Roche) and DNase I (20 μg/mL; MilliporeSigma) for 30 minutes in a 37°C water bath with occasional agitation. Digested tissue was passed through a 70 μm cell strainer to isolate the cells, and these cells were washed with PBS.

### Single-cell RNA-Seq using Seq-Well S^3^.

After obtaining single-cell suspensions from fresh biopsies, we used the Seq-Well S^3^ platform. Full methods on implementation of this platform are described (23, 24). Briefly 15,000 cells in 200 mL RPMI with 10% FBS were loaded onto a PDMS array preloaded with barcoded mRNA capture beads (ChemGenes) and settled by gravity into distinct wells. The loaded arrays were washed with PBS and sealed using a polycarbonate membrane with a pore size of 0.01 μm, which allows for exchange of buffers but retains biological molecules within each nanowell. Arrays were sealed in a dry 37°C oven for 40 minutes and submerged in a lysis buffer containing guanidium thiocyanate (MilliporeSigma), EDTA, 1% β-mercaptoethanol, and sarkosyl (MilliporeSigma) for 20 minutes at room temperature. Arrays were transferred to hybridization buffer containing NaCl (Thermo Fisher Scientific) and supplemented with 8% (v/v) polyethylene glycol (PEG, MilliporeSigma) and agitated for 40 minutes at room temperature. mRNA capture beads with mRNA hybridized were collected from each Seq-Well array, and beads were resuspended in a master mix for reverse transcription containing Maxima H Minus Reverse Transcriptase (Thermo Fisher Scientific EP0753) and buffer, dNTPs, RNase inhibitor, a 50 template switch oligonucleotide, and PEG for 30 minutes at room temperature, and overnight at 52°C with end-over-end rotation. Exonuclease I treatment was used (New England Biolabs [NEB] M0293L) to remove excess primers. After exonuclease digestion, bead-associated cDNA was denatured for 5 minutes in 0.2 mM NaOH with end-over-end rotation. Next, beads were washed with TE buffer + 0.01% Tween-20, and second strand synthesis was carried out by resuspending beads in a master mix containing Klenow Fragment (NEB), dNTPs, PEG, and the dN-SMRT oligonucleotide to enable random priming off the beads. PCR amplification was carried out using KAPA HiFi PCR Mastermix (Kapa Biosystems KK2602) with 2.00 beads per 50 μL reaction volume. After whole transcriptome amplification, libraries were pooled in sets of 6 (12.000 beads) and purified using Agencourt AMPure XP SPRI beads (Beckman Coulter, A63881) by a 0.6× volume ratio, followed by a 0.8×. Libraries’ size was analyzed using an Agilent Tapestation high sensitivity D5000 kit (Agilent Technologies) with an expected peak at 1000 bp and absence of smaller primer peaks. Libraries were quantified using Qubit High-Sensitivity DNA kit and preparation kit, and libraries were constructed using Nextera XT DNA tagmentation (Illumina FC-131-1096) using 800 pg of pooled cDNA library as input using index primers with format as done before (24). Amplified final libraries were purified twice with AMPure XP SPRI beads as before, with a volume ratio of 0.6× followed by 0.8× yielding library sizes with an average distribution of 650–750 bp. Libraries from 16 Seq-Well arrays were pooled and sequenced together using an Illumina NovaSeq 6000 S2 Reagent Kit v1.5 (100 cycles) using a paired-end read structure with custom read 1 primer: read 1: 20 bases with a 12-base cell barcode and 8-base UMI; read 2: 82 bases of transcript information, index 1 and index 2: 8 bases.

### Single-cell RNA-Seq computational pipeline and analysis.

Raw sequencing data were converted to demultiplexed FASTQ files using bcl2fastq2 based on Nextera N700 indices corresponding to individual arrays. Reads were then aligned to hg19 genome assembly and aligned using the Dropseq-tools pipeline on Terra (https://app.terra.bio). Data were normalized and scaled using Seurat R package v.3.1.0 (https://satijalab.org/seurat/); any cell with fewer than 750 UMIs or greater than 2500 UMIs was excluded from further analyses. This cells-by-genes matrix was then used to create a Seurat object. Cells with any gene expressed in fewer than 5 cells were discarded from downstream analysis, and any cell with at least 300 unique genes was retained. Cells with more than 20% of UMIs mapping to mitochondrial genes were then removed. These objects were then merged into 1 object for pre-processing and cell type identification. The combined Seurat object was log-normalized to UMI+1 using a scale factor of 10,000. We examined highly variable genes across all cells, yielding 2000 variable genes. Principal component analysis was applied to the cells to generate 100 principal components (PCs). Using the JackStraw function within Seurat, we identified significant PCs to be used for subsequent clustering and further dimensionality reduction. For 2D visualization and cell type clustering, we used a UMAP dimensionality reduction technique and with “min_dist” set to 0.5 and “n_neighbors” set to 30. To identify clusters of transcriptionally similar cells, we employed unsupervised clustering as described above using the FindClusters tool within the Seurat R package with default parameters and k.param set to 10 and resolution set to 0.5. We applied the default parameters with a shared nearest neighbor parameter optimized for each combined data set inside Monocle 3 package (V3.2.0) to construct single-cell pseudo-time trajectory to discover differential transitions. We used highly variable genes identified by Seurat to sort cells in pseudo-time order. The actual precursor determined the beginning of pseudo-time in the first round of “orderCells.” UMAP was applied to reduce dimensional space, and the minimum spanning tree on cells was plotted by the visualization function “plot_cells” for Monocle 3. To further characterize substructure within cell types (for example, epithelial cells), we performed dimensionality reduction (PC analysis) and clustering over those cells alone. Differential expression analysis between the negative and positive groups of the same cell type was performed using the Seurat package FindAllMarkers in Seurat v3 (setting “test.use” to bimod). For each cluster, DEGs were generated relative to all the other cells. GO, gene set enrichment, and Kyoto Encyclopedia of Genes and Genomes pathway analyses from DEGs were performed using Metascape (https://metascape.org), which supports statistical analysis and visualization profiles for genes and gene clusters.

### Histology and multicolor fluorescence immunohistochemistry.

Formalin (4%) fixed duodenum and colon tissue samples were embedded in paraffin, and a 4 μm section of each was obtained on a glass slide. These sections were deparaffinized and incubated with anti-HIV p24 (clone: Kal-1, Dako), anti–SARS-CoV-2 nucleoprotein (clone: 40143-T-62, Sino Biological) anti-ACE2 (clone: ab15348; Abcam), anti-TMPRSS2 (clone: ab109131; Abcam), and anti-EpCAM (clone: ab71916; Abcam) followed by a secondary antibody incubation using the Opal 4-color manual IHC (PerkinElmer) as instructed by the manufacturer. For Opal fluorophores (PerkinElmer), FITC (product number FP1487001KT; PerkinElmer) was used for EpCAM and p24, Texas red (product number FP1488001KT; PerkinElmer) for ACE2, and Cy5 (product number FP1497001KT; PerkinElmer) for TMPRSS2 and SARS-CoV-2 nucleoprotein signal generation. DAPI was used as the nuclear counterstain. The sections were mounted with the Fluorescence Mounting Medium (catalog number S302380-2; Agilent Technologies) and cover-slipped, and the edges were sealed with nail polish. The slides were stored at 2°C–8°C until images were acquired.

### Microscopy and quantitative image analysis.

Images of the tissue sections were acquired using the TissueFAXS software (TissueGnostics) connected with a Zeiss Axio Observer Z1 inverted microscope (Olympus). The quantitative analysis of the cells of the different phenotypes within the images was done using the TissueQuest quantitation software (TissueGnostics).

### Data availability.

Next-generation sequencing data were deposited in National Center for Biotechnology Information’s Gene Expression Omnibus under accession number GSE181877.

### Statistics.

Graphs were plotted using Prism 8.4.3 (GraphPad Inc.). Differences between groups were analyzed using the Seurat package FindAllMarkers in Seurat v3 (setting ‘‘test.use’’ to bimod). If any other specific test was used, it has been stated in the figure legends. A *P* value less than 0.05 was considered significant.

### Study approval.

This study was approved by the Biomedical Research Ethics Committee of the University of KwaZulu-Natal (BE 021/13 and BE061/13). Participants gave written informed consent.

## Author contributions

RF and OEA performed experiments and analyzed transcriptional data. SN supervised transcriptional analysis. NH, YZ, AS, AN, and IMM contributed to experimental work. NM consented participants and collected samples. DR and FK coordinated human tissue sample collection. WK, FGM, VTM, and FA contributed surgical human tissue samples. BB, THP, and AKS supervised data analysis. AKS, AL, and HNK provided intellectual input. RF, OEA, AL, and HNK prepared the manuscript. HNK conceptualized and supervised the work.

## Supplementary Material

Supplemental data

Supplemental table 1

Supplemental table 2

Supplemental table 3

## Figures and Tables

**Table 1:**
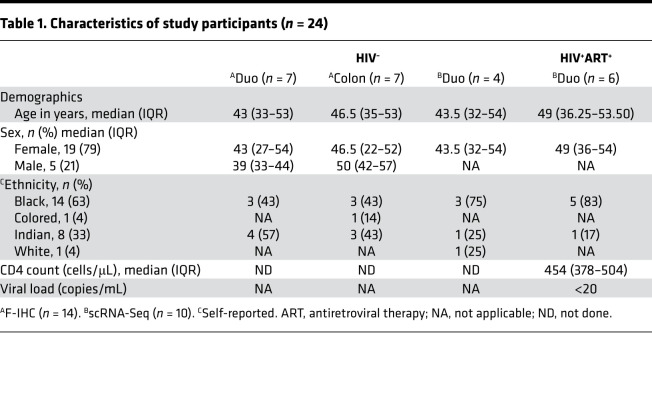
Characteristics of study participants (*n* = 24)

## Version 1 07/12/2021

In-Press Preview

## Version 2 08/23/2021

Electronic publication
