# Supplementary material for: HIV infection drives interferon signaling within intestinal SARS-CoV-2 target cells
Source: JCI Insight. 2021 Aug 23;6(16):e148920. doi: 10.1172/jci.insight.148920 (PMC8409978; doi:10.1172/jci.insight.148920)
Supplement: Supplemental data [file jciinsight-6-148920-s200.pdf]

Supplementary information

Supplemental information includes 6 Figures and 3 Tables

Figure S1-S6

Table S1 Individual study participant information

Table S2A List of differentially expressed genes defining cell type in duodenum

Table S2B List of differentially expressed genes upregulated in ACE2+ Epithelial Cells vs. ACE2- Epithelial Cells from scRNA-seq.

Table S2C List of differentially expressed genes upregulated in ACE2+/TMPRSS2+ Remaining Epithelial vs. All Remaining Epithelial

Table S2D Predicted upstream drivers of DE response between ACE2+ Epithelial cells and ACE2- Epithelial cells from duodenum

Table S3A Statistics for ACE2 and TMPRSS2 enrichment in duodenum epithelial cells

Table S3B List of differentially expressed genes upregulated in HIV+ Absorptive Cells vs HIV- Absorptive Cells

Table S3C List of differentially expressed genes upregulated in ACE2+HIV+ Absorptive Cells vs ACE2+HIV- Absorptive Cells

Table S3D Statistics for Genes Significantly Upregulated in ACE2+HIV+ (ART) Absorptive Cells vs ACE2+HIV- Absorptive Cells

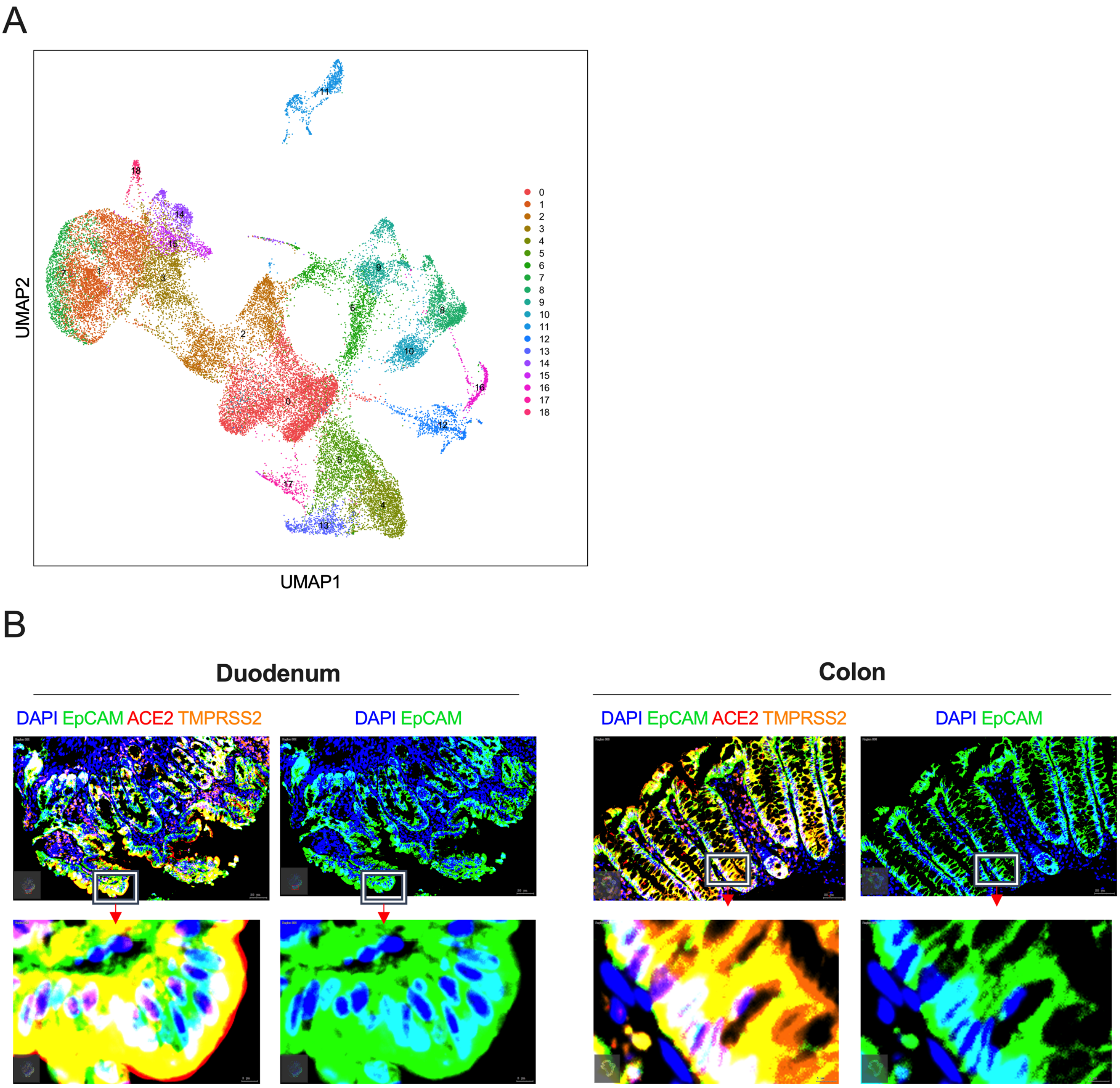

Figure S1 - related to Figure 1

A. UMAP of 32381 cells, identifying 18 different clusters **B.** Representative fluorescent immunohistochimestry image of gut tissue showing *ACE2* (red), *TMPRSS2* (orange), EpCAM (green) and DAPI (blue) of duodenum and colon. Bars, 20 μm for all images.

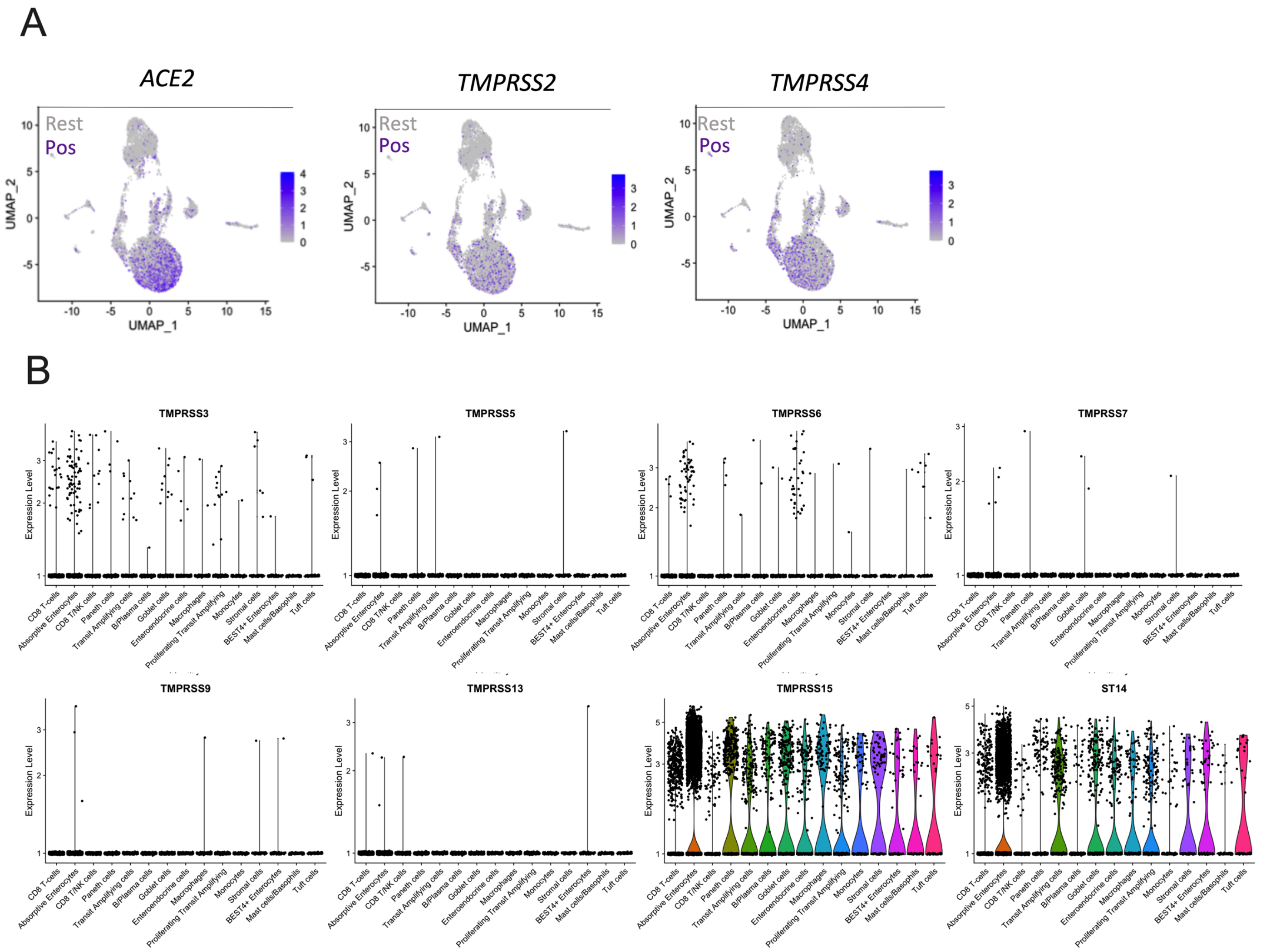

Figure S2 - related to Figure 2

A. UMAP projection of all cells from duodenal biopsises with points coloured by detection of *ACE2* (left), *TMPRSS2* (middle) and *TMPRSS4,* (left). Colour coding is as follows: purple, RNA positive, grey, RNA negative with 'Rest' referring to cell not expressing the indicated marker. **B.** Violin plot of different serine protease expression in the duodenum.

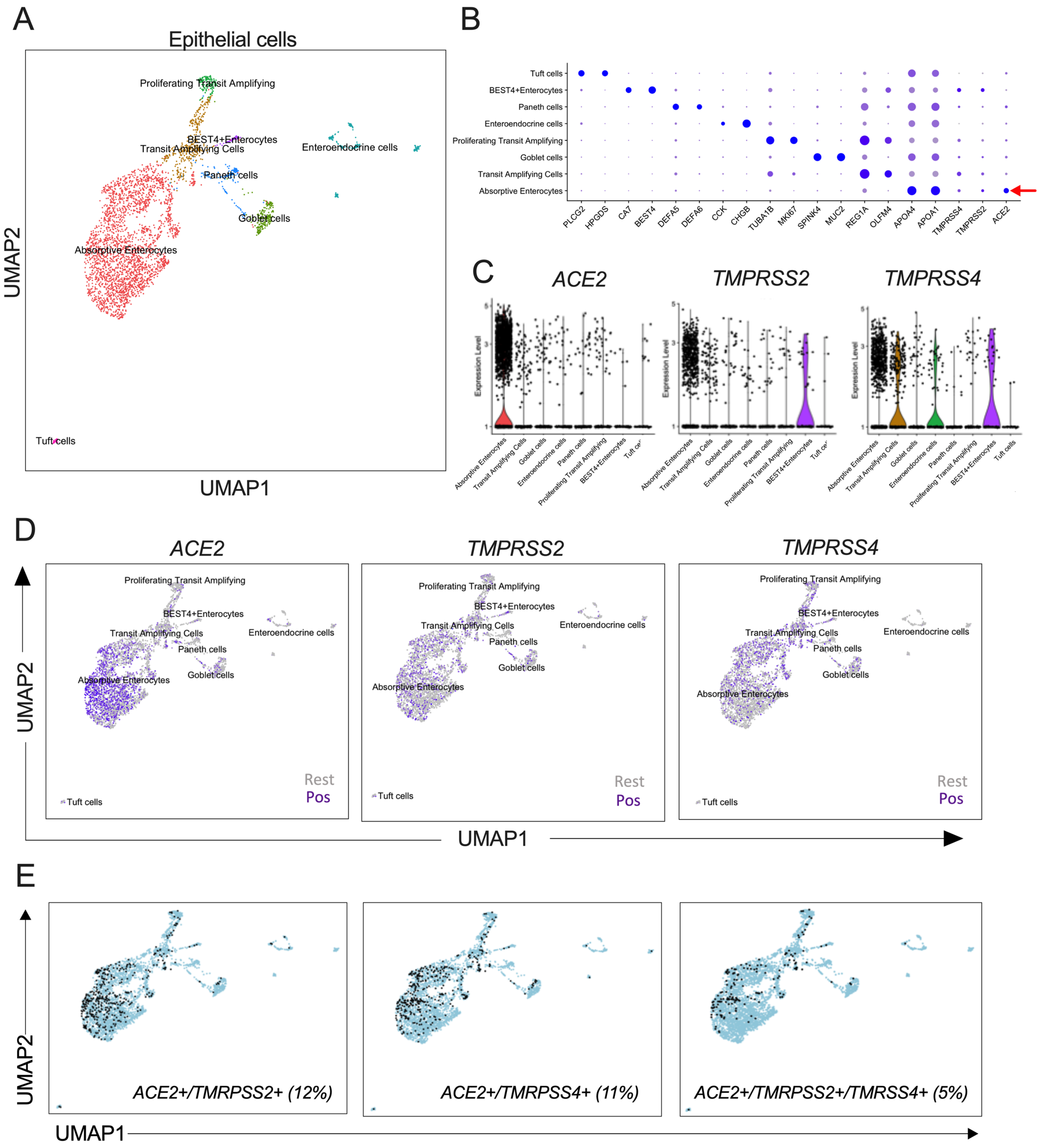

Figure S3 - related to Figure 2

A. UMAP of 5927 epithelial cells coloured by cellular subsets **B.** Dot plot of 2 defining genes of each cell type, with *ACE2*, *TMPRSS2* and *TMPRSS4.* Dot size represents fraction of cells within cell type expressing a given gene, and colour intensity represents binned count-based expression amounts (log(scaled UMI+1) among expressing cells. Red arrow indicate cell type with largest proportion of *ACE2+TMPRSS2+TMPRSS4+* cells. **C.** Expression of *ACE2* (left), *TMPRSS2* (middle) and *TMPRSS4* (right) among all epithelial subsets from duodenum. **D.** UMAP projection as in (A) but with overlay of cells expressinng *ACE2* (left), *TMPRSS2* (middle) and *TMPRSS4* (right) colour coded as purple=RNA positive and grey=RNA negative with ‘Rest’ referring to cells not expressing the indicated marker. **E.** UMAP protection of cells co-expressing *ACE2+TMPRSS2+* (left), *ACE2+TMPRSS4+* (middle) and *ACE2+TMPRSS2+TMPRSS4+* cells (right) among all epithelial cells.

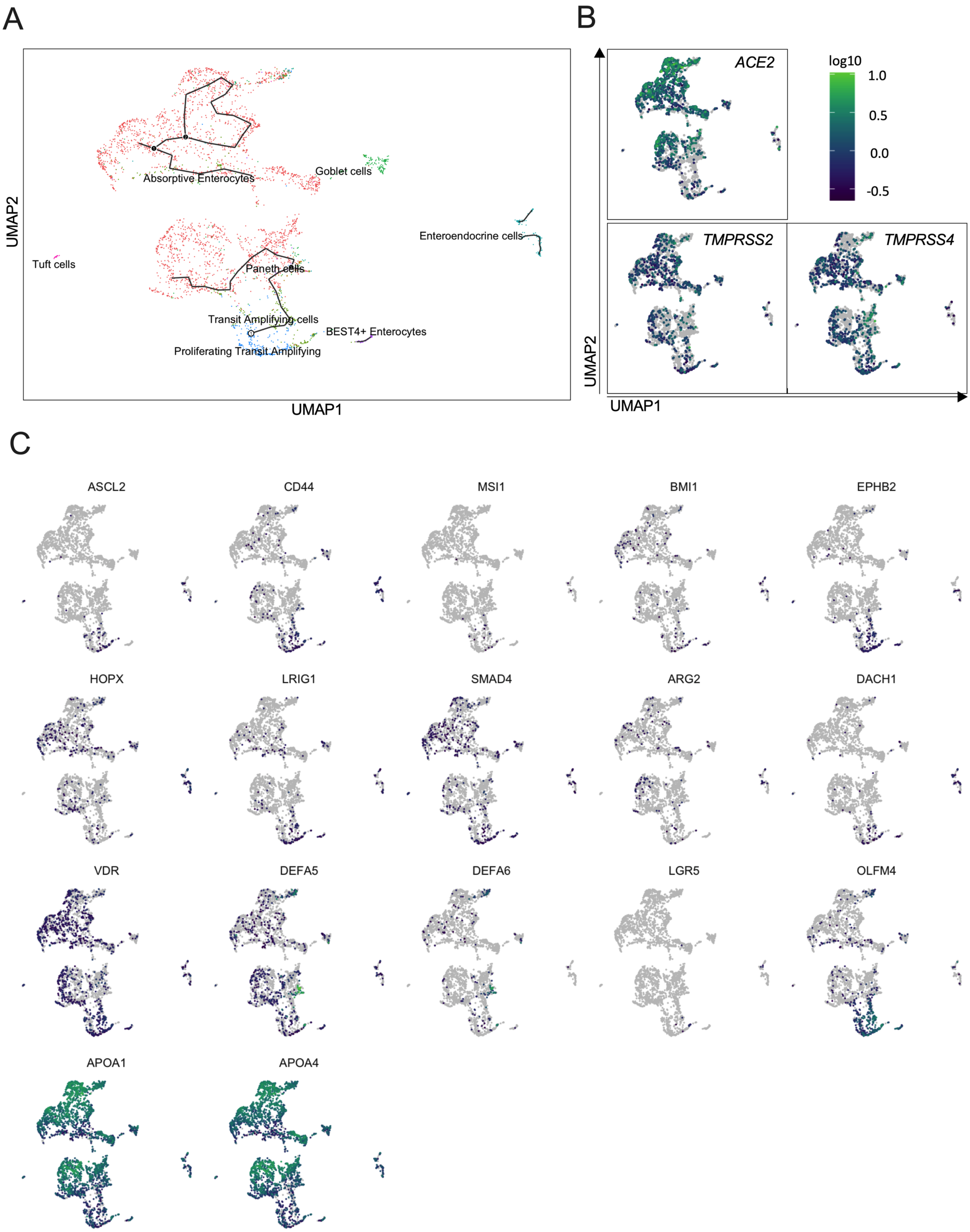

Figure S4 - related to Figure 2

A. UMAP plot showing genes specifically expressed in intestinal stem cell on the monocle map, showing different stages of epithelial cell differentiation. **B.** Monocle map showing the overaly of *ACE2, TMPRSS2* and *TMPRSS4* expression on to the monocle map in A. **C.** Monocle map show overlay of indated gene expression relevant for cellular identification shown in A.

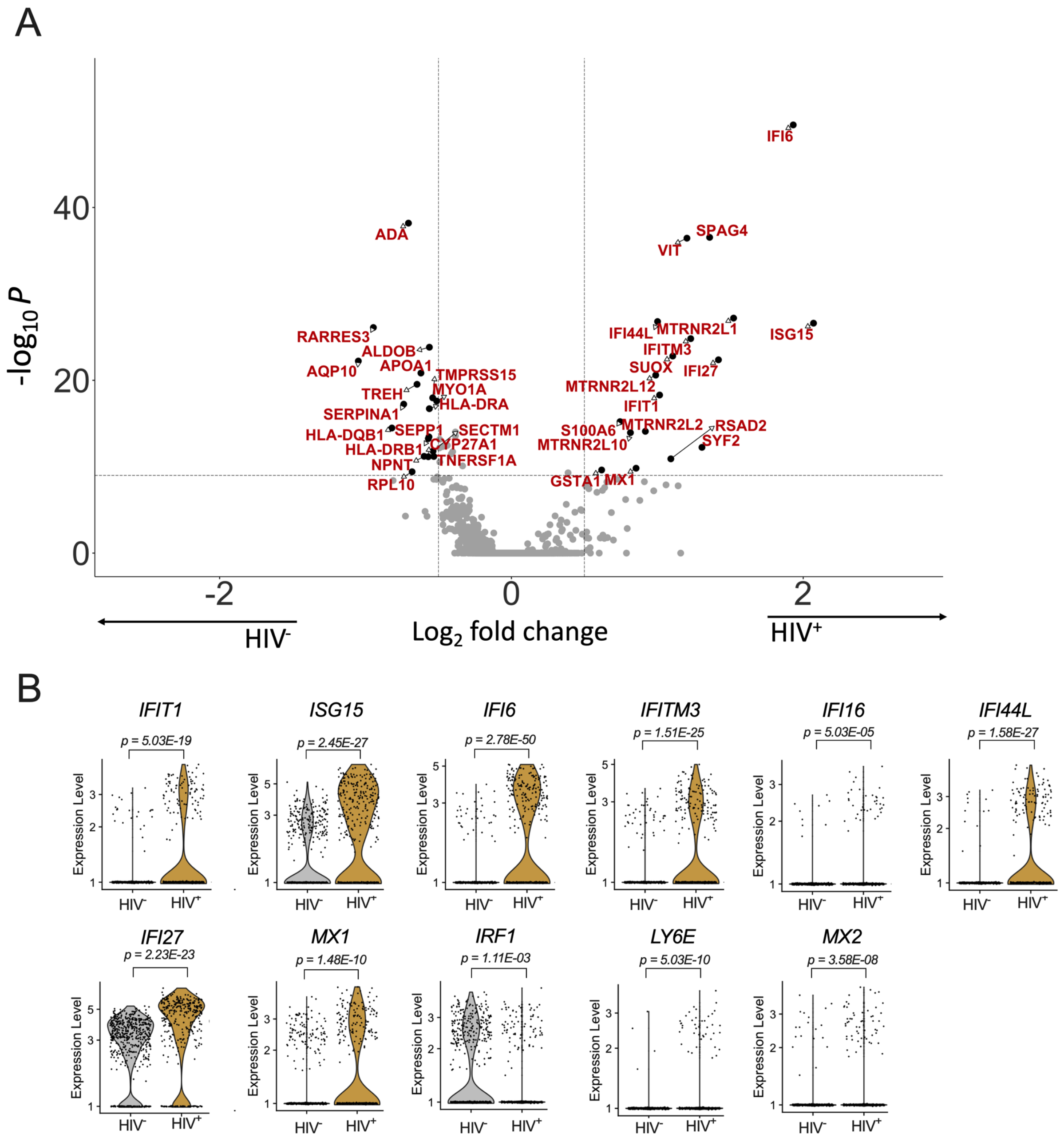

Figure S5 - related to Figure 3

A. Volcanao plot of DEGs within absorptive enterocytes expressing ACE2 comparing HIV uninfected (n=4) and HIV infected individuals (n=5) and highlighting genes in red with more than 0.5 fold change and adjusted P-values Padj < 5.0E10-08. Genes differentially expressed among *ACE2* expressing absorptive enterocytes comparing HIV uninfected and HIV infected individuals. FDR-adjusted $p < 0.05$; full results can be found in Table S3C).

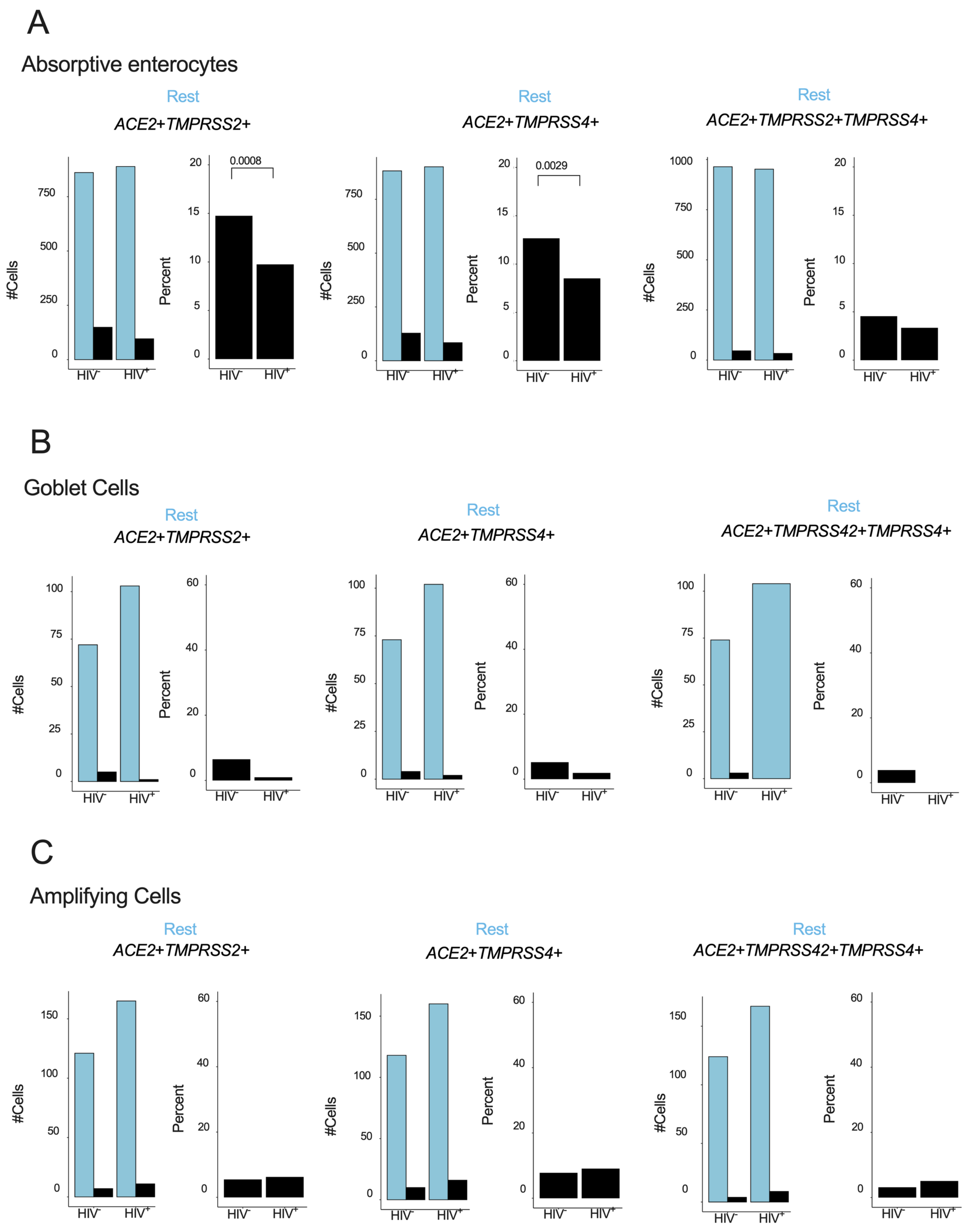

Figure S6 - related to Figure 4

Actual number of cells (left) and % (right) of co-expressing *ACE2+TMPRSS2+*, *ACE2+TMPRSS4+* and *ACE2+TMPRSS2+TMPRSS4+* positive cells by HIV status for **A.** Absorptive enterocyts, **B.** Goblet cells and **C**. Transit Amplifying. *P*-values by Fisher's Exact Test.
